# A Simple Method for Polymer Screening for Selection of Food Packaging Material

**DOI:** 10.3390/polym18192426

**Published:** 2026-10-05

**Authors:** Costas Tsioptsias, Konstantina Konstantopoulou, Karmen Nanakou

**Affiliations:** Department of Food Science and Technology, Alexandrian University Campus at Sindos, International Hellenic University, 57400 Thessaloniki, Greece; konstantopoulouk3@gmail.com (K.K.); karmennanakou22@gmail.com (K.N.)

**Keywords:** Hansen solubility parameters, migration, permeability, glass transition, storage temperature, maximum exposure temperature

## Abstract

The requirements and specifications of food packaging materials are continuously increasing, e.g., due to new regulations. Selection of a proper material is a multi-criteria decision, and various aspects must be considered. In this work, a simple methodology is proposed for polymer screening for food packaging applications. The methodology accounts for migration risk and permeability to oxygen and volatile flavor compounds based on the Hansen Solubility Parameters (HSPs). In addition, the glass transition temperature (T_g_), as well as the storage temperature of the food and the maximum exposure temperature of the material (e.g., in the case of hot filling), are taken into account. Based on these data, for a given food and for each polymer, five sub-scores are calculated. For a given food, 22 polymers are ranked according to their total score, which is derived as the sum of the five sub-scores. However, the final material selection is not based solely on the total score, since the sub-scores provide insights regarding the specific advantages and disadvantages of each polymer to be used as packaging for a given food. The proposed methodology can assist in recognizing proper candidate polymers, exclude polymers that for a given food would be clearly unsuitable and even design multi-layered composites with improved properties. One of the basic assumptions of the proposed methodology is that food–polymer interactions are important for assessing migration risk. Migration tests in two different food simulants were used for a preliminary support of this assumption.

## 1. Introduction

Food packaging is essential for the preservation of food quality and shelf life. Metals [1], ceramics [2], polymers (including biopolymers) [3] and composite materials can be used for food packaging [2]. Among these materials, polymers or polymer composites are perhaps the most popular. Polymeric materials in general are characterized by low weight, low cost, easiness and versatility in processing and shaping and good mechanical properties. Despite these advantages, polymers, compared to glass/ceramics and metals, exhibit rather poor barrier properties. Additionally, during polymer processing, various additives and substances with low molecular weight are used, e.g., plasticizers, antioxidants, pigments, catalysts, monomers, solvents, etc. All these are potential migrants from packaging into food [4,5]. Migration is considered a major issue for the environment and human health, and for this reason, in the past years, new regulations and legislations have been published, e.g., the 10/2011 European Commission Regulation regarding plastic materials and articles intended to come into contact with food [6].

The new regulations inevitably result in increased and specialized specifications that must be fulfilled by candidate food-contact materials. Quality assurance has led to a continuously growing interest in smart/active packaging materials such as materials with temperature and/or humidity sensors, controlled release and migration of desired compounds, etc. [7,8]. Of course, such smart materials should also fulfill the “traditional” specifications and requirements, such as low permeability (for oxygen but also volatile flavor compounds of the food) [9], good mechanical performance, opacity or transparency depending on application, low migration, proper thermal transition temperatures, etc. Despite the desired and controlled interaction between packaging material and food that exists in active packaging materials, in general, the packaging material, ideally, should be inert with respect to the food. A strong interaction between food or food components and packaging material may lead to local plasticization-related effects on the polymer. Such effects are the increase in the free volume of the polymer and the increase in the polymer’s chain mobility [10]. These effects favor diffusion, which is the primary mechanism for migration. For the same reason, exposure of the packaging material to temperatures near or above the polymer’s glass transition temperature (T_g_), e.g., in the case of hot filling, besides possibly having a severe impact on mechanical performance, will also facilitate migration. Thus, both the exposure conditions of the polymer and its T_g_ are important factors for both mechanical performance and migration.

Based on the above, it seems that for a given food, the selection of a proper packaging material is a difficult decision, which should be made by taking into accounts several aspects, including the conditions of packing exposure (e.g., temperature), the T_g_ of the polymer, the permeability of the material not only to oxygen but also to flavor compounds from the specific food, as well as the migration risk. To the best of the authors’ knowledge, the relative literature directly dealing with this question is rather limited [11]. Various models have been proposed but are highly specific, e.g., models and simulations tools solely focusing on oxygen permeation [12] or modified atmosphere packaging [13], as well as the time evolution of O_2_ and CO_2_ in the headspace of packaging. Clearly, such models and simulations are useful but cannot fully answer the question: Which material is the best for a given food? As mentioned above in one article, this is the main question of interest [11]. In the proposed decision-making tool, various aspects are taken into account, such as humidity and temperature of storage, the T_g_ of the polymer, O_2_ permeation and CO_2_ for modified atmosphere applications and others [11]. Although these are very important aspects that should be taken into account for the selection of the proper packaging material, two important aspects have not been considered: a) the permeation of volatile flavor compounds and b) the food–polymer interactions and the migration risk. The proposed tool [11] is heavily based on available data. For the permeation of common vapor/gas molecules like O_2_, CO_2_ and H_2_O, adequate experimental data exist for various polymers. However, for flavor compounds, e.g., cinnamaldehyde (mainly responsible for the odor of cinnamon) it is unlikely that permeability data exist for any polymer. In addition, such flavor compounds may number in the hundreds, and it is clear that experimental permeability data for the various combinations of flavor compound–polymer should be extremely limited.

Hansen solubility parameters (HSPs) are parameters characteristic of every substance. They are composed of three partial contributions: the dispersion HSP, the polar HSP and the hydrogen bond HSP, respectively, accounting for the potential of a molecule to form dispersion forces, polar interactions and hydrogen bonds. The comparison of the HSPs of two substances allows for the prediction of existence or absence of favorable interactions, and thus, the HSPs can be used to predict solubility/compatibility between two substances [9,14]. HSPs are widely used for solvent screening in a broad range of applications, e.g., for finding solvents with low toxicity [15], for the extraction of a specific compound [16], for lignocellulosic biomass fractionation [17], as well as for extraction for analytical purposes [18,19]. Besides solvent screening, HSPs have been used to predict interactions between two substances for various purposes, including polymer–foulant interactions for predicting fouling of membranes used in wastewater and water treatment [20], polymer permeability for predicting flavor scalping [9], as well as migration from packaging material [21]. Briefly, HSPs can be very useful as a rapid and easy-to-apply tool for predicting permeability and migration without requiring existing experimental data. Of course, permeability and migration are quite complex phenomena that depend on various factors, such as diffusion, crystallinity, free volume, etc. Such factors cannot be taken into account by HSPs.

Based on the above, the scope of this study is to develop a simple method for the selection of proper food packaging material for a given food. The proposed methodology is heavily but not exclusively based on HSPs The use of HSPs introduces two main elements of novelty and, specifically, it allows us to account for migration risk and the permeability of flavor compounds. Such issues are not commonly considered in other decision-supported approaches due to lack of appropriate data; e.g., permeability values of CO_2_ or O_2_ for various polymers are known, but such data are limited for flavor compounds. Besides the migration risk and the permeability to volatile flavor compounds, the permeability to oxygen, the T_g_ of the polymer, the storage temperature and the maximum exposure temperature of the packaging are considered. For a given food, various polymers are examined and ranked based on the above-mentioned aspects. The basic assumption of the proposed methodology is that food–polymer interactions are an indirect indicator of migration risk. This hypothesis was experimentally tested by carefully designed migration experiments.

## 2. Materials and Methods

### 2.1. Experimental

Poly(vinyl alcohol) (PVA) raw powder (M_w_ = 89,000–98,000 g/mol and >99% hydrolyzed) was purchased from Sigma Aldrich (St. Louis, MO, USA). Polystyrene (PS) pellets (M_w_ = ~100,000 g/mol) were purchased from BDH limited (Poole, UK). Chloroform (>99.8%) was purchased from Fisher Chemical (Pittsburgh, PA, USA). Olive oil was purchased from the local market.

About 1 g of PS was dissolved in 10 mL of chloroform by magnetic stirring at room temperature for 30 min. After the dissolution of the polymer, 50 μL of olive oil was added to the solution, and the mixture was magnetically stirred for 5 min to ensure the full mixing of the olive oil with the polymer solution. The solution was cast in a petri dish and was left at room temperature for 72 h to ensure solvent evaporation and PS film formation.

An oil-in-water emulsion was formed by mixing 50 mL of distilled water with 250 μL of olive oil. The mixture was ultrasonicated at a power of 500 W/L for 15 min using a Sonopuls HD ultrasound device (Bandelin, Germany). About 1 g of PVA was dissolved in 10 mL of the emulsion by magnetic stirring and heating at around 75 °C for 20–30 min. The PVA solution was cast in a petri dish and left at room temperature for 72 h for film formation to occur. The films were stored at room conditions, and no other special conditioning was applied to the films.

Pieces from the PS-olive oil and PVA-olive oil films were immersed in 50 mL of 10% (*v*/*v*) ethanol aqueous mixture (simulant A) and 50% (*v*/*v*) ethanol aqueous mixture (simulant D1) for 24 h at room temperature. These are two of the five food simulants that are proposed in the EU regulation for plastic materials and articles in contact with food [6]. The thickness of the films was measured with a digital caliper (± 0.01 mm). After immersion, the films were air-dried at room temperature for 24 h. The mass of the films before and after immersion was measured with a scale (± 0.0001 g). To ensure complete removal of the simulants, the mass of the films was also measured after the films had been kept for 2 h at 40 °C. Non-immersed films were used as the first reference/blank samples. No mass loss was observed in the immersed and non-immersed samples after heating at 40 °C. All experiments were performed twice (two replicates) by using two different pieces from the same film. The film thicknesses used in the experiments are presented in Table 1. Using exactly the same procedure and concentrations, neat PS and PVA polymer films (without olive oil) were prepared as second reference samples (two replicates from pieces of the same film). Their thicknesses are also presented in Table 1. The extent of migration from a film with a thickness of 1 mm was expressed as % mass loss:

(1) $$migration=100\times \frac{initial\;mass-final\;mass}{initial\;mass}\times \frac{actual\;film\;thickness\;in\;mm}{1\;mm}$$

Two explanations will be provided for the multiplication of the % mass loss ($100\frac{initial\;mass-final\;mass}{initial\;mass}$) with actual film thickness. One is based on mathematics, and the other is based on Fick’s First Law. If migration was proportional to the film thickness, then if we wanted to normalize/express the migration for 1 mm thickness, then the migration would be divided by the actual thickness. For example, for a 0.2 mm film, we would write the rule of three as follows (if migration was proportional to actual film thickness):

At a thickness of 0.2 mm, the migration is 3%.

For a 1 mm thick film, the migration is x.

⇒x=1×3%/0.2

However, migration is expected to be inversely proportional to film thickness or, in other words, migration is proportional to 1/(actual thickness). Thus, if the simple rule of three is applied, equation 1 is derived. As can be seen in Table 1, in all polymer–simulant pairs, the difference in the thickness in the two replicates was small, with the exception of the PVA–simulant A pair. As can be seen in the second replicate, the thickness was twice (0.44 mm) that of the first replicate (0.22 mm). According to Fick’s first law of diffusion, the diffusion rate is inversely proportional to the film thickness. Thus, the migration rate in the thicker film (0.44 mm) will be half that in the other film (0.22 mm). The migration (diffusion) rate influences the final mass. Consequently, the term $\frac{initial\;mass-final\;mass}{initial\;mass}$ was different in the two replicates (while in all other cases, the two replicates yielded similar values). According to Fick’s first law, the diffusion rate multiplied by the thickness, Δx, is constant and equal to DxΔC (where D is the diffusion coefficient and ΔC is the concentration difference). In other words, the term $\frac{initial\;mass-final\;mass}{initial\;mass}$ in the case of the thicker film (0.44 mm) was lower than that of the other film (0.22 mm) because the thickness was higher, and thus the migration rate was lower. However, if the term $\frac{initial\;mass-final\;mass}{initial\;mass}$ for the actual thickness (0.22 or 0.44 mm) is multiplied by the real thickness, it should be constant and equal to the respective term for 1 mm (this is a direct consequence of Fick’s first law). Indeed, after multiplication, the migration was quite similar for the two films. It should be stressed that the units of migration in Equation 1 are % w/w, and no length units are involved.

### 2.2. Theoretical Methodology

The polymer screening of various candidate materials for a given food is based on the classification of the polymers according to a total score. The total score is calculated from 5 sub-scores. For the calculation of the first two sub-scores, the HSPs of the food are needed. The HSP values of the food were calculated from the following established equations for estimating the HSP of mixtures [9,14]:

(2) $$\delta_{d,food}=x_{1}\times \delta_{d,1}+x_{2}\times \delta_{d,2}+\cdots +x_{n}\times \delta_{d,n}$$

(3) $$\delta_{p,food}=x_{1}\times \delta_{p,1}+x_{2}\times \delta_{p,2}+\cdots +x_{n}\times \delta_{p,n}$$

(4) $$\delta_{hb,food}=x_{1}\times \delta_{hb,1}+x_{2}\times \delta_{hb,2}+\cdots +x_{n}\times \delta_{hb,n}$$

where

δ_d,n_: dispersion partial HSP of the n-th component;

δ_p,n_: polar partial HSP of the n-th component;

δ_hb,n_: hydrogen bond partial HSP of the n-th component;

x_n_: the volume fraction of the n-th component.

The volume fractions of the food were estimated from the nutritional statement value (content in fats and lipids, carbohydrates and proteins). The salt was neglected since its HSP values are not known, while the remainder was considered to be water. Densities of 0.92 g/mL, 1.3 g/mL and 1.5 g/mL were considered, respectively, for fats, proteins and carbohydrates. The conversion of nutritional statement values to volume fractions was carried out as follows: a basis of 100 g of food was used. The mass of each component was divided by the respective density in order to convert the mass to volume. Then, the volumes of all components were added in order to calculate the total volume, and the volume fraction of each component was calculated by dividing the respective volume by the total volume.

For the HSP values of carbohydrates, proteins and lipids, the average values of various respective components that were presented previously [21] were used. The HSP values of various polymers were retrieved from the literature [9]. The distance in Hansen space, R_a_, between two substances is defined as follows [9,14]:

(5) $$R_{a}=\sqrt{4{\left(\delta_{d1}-\delta_{d2}\right)}^{2}+{\left(\delta_{p1}-\delta_{p2}\right)}^{2}+{\left(\delta_{hb1}-\delta_{hb2}\right)}^{2}}$$

High values of R_a_ (typically > 8) indicate low compatibility between the two substances. On the contrary, compatible/miscible substances exhibit low values of R_a_. For a given food, the R_a_ between the food and the various candidate polymers was calculated using Equation 5. Then, these values were normalized by dividing them by the maximum R_a_ that was observed. In this way, each polymer receives its score1:

(6) $$score1=\frac{R_{a_{polymer-food}}}{{\left(R_{{a,}_{polymer-food}}\right)}_{max}}$$

Score1 reflects the potential interaction between the polymer and the food. Obviously, the lowest possible interaction is desired. A strong interaction between the food and the packaging material can lead to effects similar to those that occur during polymer plasticization, i.e., an increase in the polymer’s free volume, an increase in chain mobility, etc. All of these effects are expected to favor and enhance migration. In addition, permeability may also be increased, mechanical properties may be affected, etc. Thus, the polymer which exhibits the highest R_a_ for a given food (minimum compatibility) must receive the highest score1. For this reason, the values were normalized by the maximum observed value of R_a_. The polymer responsible for this maximum value will receive a score1 of 1, while other polymers, depending on their R_a_ values for the food, will receive a proportionally lower score1.

Since many foods are complex dispersions and not solutions, score 2 was calculated as follows in order to provide a second additional estimate of the potential interaction between the food and the polymer:

(7) $$score2=\frac{{\sum x}_{{Nutrient}_{i}}\times R_{a_{{Polymer-Nutrient}_{i}}}}{{\left({\sum x}_{{Nutrient}_{i}}\times R_{a_{{Polymer-Nutrient}_{i}}}\right)}_{max}}$$

where

$x_{{Nutrient}_{i}}$: the volume fraction of ith nutrient (proteins, carbohydrate etc.).

As mentioned above, score1 and score2 can indirectly provide insights into the risk of migration. Score3 accounts for the probability of permeation of the polymer by volatile flavor compounds and oxygen. From the R_a_ between a volatile compound and a polymer, the probability of permeation can be estimated [9]. The HSP values of flavor compounds and oxygen were retrieved from the literature [9]. Score3 is calculated similarly to score1 and score2. For a given food, the primary compounds responsible for the flavor are first identified. Then, for each of these compounds, as well as oxygen, the R_a_ distances from the polymers are calculated and normalized with respect to the maximum observed R_a_ value in order to obtain sub-score3_i:_

(8) $${score3}_{i}=\frac{R_{a_{polymer-flavor,i}}}{{\left(R_{a_{polymer-flavor,i}}\right)}_{max}}$$

Then, score 3 is calculated as the average of the various sub-score3_i_ values, along with the respective standard deviation (Devscore3):

(9) $$score3=Average\;({score3}_{i})$$

(10) $$Devscore3=Standard\;deviation\;({score3}_{i})$$

Devscore3 can provide insights into the permeability of the polymer to all substances of interest. If the permeation of all substances is similar (either high or low), then Devscore3 will be low. In contrast, if a polymer has low permeability to oxygen but high permeability to a flavor compound, then Devscore3 will be high. Thus, by taking into account Devscore3, it can be readily determined whether the permeability of all substances of interest is similar.

Besides migration and permeation, the proposed methodology accounts for the storage temperature (T_storage_) of a given food, as well as the maximum temperature (T_processing_) to which the packaging material will be exposed when in contact with the food. For example, in the case of hot filling a packaging material with food at a temperature of 70 °C, T_processing_ will be 70 °C. Otherwise, it will be the same as T_storage_. The glass transition temperature of the polymer is compared with T_storage and_ T_processing_ and score4 and score5, respectively, are calculated from the following equations:

(11) $$score4=\frac{T_{g}-T_{storage}}{{\left(T_{g}-T_{storage}\right)}_{max}}$$

(12) $$score5=\frac{T_{g}-T_{processing}}{{\left(T_{g}-T_{processing}\right)}_{max}}$$

The total score for each candidate polymeric material is calculated from the following equation:

(13) total score=score1+score2+2×score3+score4+score5

Score1 and score2 account for migration by considering the food–polymer interactions. Specifically, score1 is obtained by comparing the HSP value of the whole food with the HSP value of the polymer, while score 2 is obtained by comparing the HSP value of each food component with the HSP value of the polymer (and by taking into account the respective volume fractions). These two approaches were used in order to ensure that the HSP values are representative and provide complementary information. Score4 and score5 account for the glass transition temperature and are influenced by it. In other words, there are two scores that are influenced by migration and two scores that are influenced by the glass transition temperature. In contrast, there is only one score for permeation (score3). Thus, if these scores are simply added, then the effect of permeability would be underestimated. Thus, score3 was multiplied by a factor of 2 in order for all scores to be given the same weight. Of course, if desired, the sub-scores can be multiplied by appropriate weighting factors. For a given food, the various candidate polymers are ranked according to their total score. However, as will be discussed in Section 3, the final decision should be made based not only on the total score but also on the understanding that the sub-scores must be taken into account.

## 3. Results and Discussion

The are two basic assumptions in the proposed methodology that could be questioned. One assumption is that foods exhibit some apparent HSP that can be estimated by treating them as mixtures. This assumption was recently tested experimentally in a previous study [22], and it was confirmed that this approach yields reasonable approximation of the HSP of foods. Specifically, the HSP of mayonnaise was experimentally evaluated and compared with that calculated from the equations for mixtures (Equations (2)–(4)) [22]. The deviation was acceptable. Mayonnaise was selected since it is one of the most complex foods. Specifically, mayonnaise is an oil-in-water emulsion, and typically, in such emulsions, water is the primary component, e.g., the water content may be 75%. However, this is not the case for mayonnaise, in which the oil content is typically higher than 70%. Thus, even for such a “peculiar” system, the mixture approach yielded acceptable approximations.

The second basic assumption of the proposed methodology is that the polymer–food interaction can indirectly provide insights into migration. This is theoretically supported by the fact that a favorable interaction of a polymer with low-molecular-weight substances can lead to effects similar to plasticization, that is, an increase in free volume and an increase in chain mobility. All these effects enable diffusion through the polymer network. Even if the interaction is not strong enough to induce an actual glass-to-rubber transition, some facilitation of diffusion would be expected. In brief, it is hypothesized that a strong interaction between a polymer and a food will enable the diffusion of various migrants and thus enhance migration. In order to test this assumption, the migration experiments (Section 2.1) on the migration of olive oil from PS and PVA films were performed. Given the limited number of polymers, simulants and replicates, these experiments provide preliminary support for the assumption and not indisputable experimental verification. The migration was tested in two different food simulants: one hydrophilic (simulant A) and one lipophilic (simulant D1) [6]. The % mass loss (total % mass loss due to migration into the simulant) along with its standard deviation is presented in Table 2. The values have been normalized by multiplication by the film thickness for reasons explained in Section 2. In Table 2, the % mass loss of the neat polymer films after immersion in the simulants is also presented. Finally, the difference between the total mass loss and the mass loss of the neat polymers is also presented in Table 2 as the % mass loss of olive oil.

As can be seen in Table 2, the behavior observed in PS and the two simulants could be explained on the basis of migrant (olive oil)–food (simulant) interactions both for overall migration and olive oil migration. Simulant D1 (50% ethanol) is more lipophilic than simulant A (10% ethanol) and thus the migration in the case of PS is higher in simulant D1 since the migrant exhibits greater solubility/affinity for simulant D1. In other words, the migration of olive oil from PS into simulants A and D1 can be explained by the migrant–food interactions. However, the other results cannot be explained on such a basis. For example, it is not clear why the migration of olive oil from PVA is higher than that from PS in simulant A. As mentioned above, simulant A is hydrophilic, and thus olive oil has the same low tendency to migrate into simulant A either from PVA or PS. We recall that the same amount of olive oil was embedded in the polymer films so as to have the same driving force for diffusion. This result can be explained on the basis of polymer–food simulant interaction. PS is of a hydrophobic nature and exhibits poor interaction with the hydrophilic simulant A. In contrast, PVA films interact strongly with and are swollen by water or aqueous solutions like simulant A. This favorable interaction enables the movement and migration of olive oil into simulant A. The increased migration from the neat PVA film compared with that from the neat PS film in simulant A can also be explained on the same basis.

Similarly, the migration from PVA in the two simulants can be explained based on the polymer–food interactions rather than migrant–food interactions. Specifically, olive oil has greater solubility (more favorable interactions) in the hydrophobic simulant D1 rather than in the hydrophilic simulant A. Thus, based on the migrant–food interaction, it would be expected that the migration of olive oil from PVA would be higher in the case of simulant D1. However, as can be seen in Table 2, the migration from PVA (both the total migration and that of olive oil) in simulant D1 was lower than that in simulant A. Again, this unexpected behavior can be explained by the polymer–food interaction. Simulant D1 can interact with PVA to a lower extent than simulant A, and thus the swelling of PVA, etc., induced by simulant D1 is lower. This hinders the diffusion and migration of olive oil into the simulant D1, resulting in a lower migration rate. Swelling is a direct consequence of polymer–simulant interactions and is important since it is associated with structural changes. Due to swelling, the free volume of the polymer is considerably increased; thus, the diffusion of migrants is enhanced. Consequently, since simulant A is more hydrophilic than simulant D1, it induces greater swelling of PVA. In addition, the incorporation of the solvent (simulant) into the polymer and the subsequent swelling can result in decreased crystallinity. Amorphous regions are commonly more mobile than crystalline regions. This, again, can enhance the diffusion of migrants. Finally, it should be mentioned that the PS–olive oil film was transparent, which suggests full miscibility of PS and olive oil (molecular level mixing). In contrast, the PVA–olive oil film was not transparent, and droplets of olive oil were dispersed in the polymer matrix. This also affects migration, but it cannot provide an explanation for the decreased migration of olive oil from PVA into the hydrophobic/lipophilic simulant D1.

The above clearly shows that, besides migrant–food interactions, polymer–food interactions are also rather important for the occurrence of migration and supports the assumption of the proposed methodology. In what follows, we present the application of the proposed methodology to three foods: (a) vinegar, which is a simple food and actually a binary solution of hydrophilic nature; (b) ketchup, which is a more complex and multicomponent food with high water content and is also typically packaged by a hot-filling process; and (c) olive oil, which is also a solution but is of a hydrophobic nature.

In Table 3, the ranking of 22 polymers for their suitability to be used as packaging materials for vinegar is presented. Before discussing the results, it should be stressed that the final selection of the optimum material cannot be based solely on the total score (the reason for this is discussed below). As can be seen in Table 3, the first two polymers (polycarbonate (PC) and poly(acrylonitrile) (PAN)) have equally high scores; however, this is for different reasons. PC exhibits the maximum possible score (1) in sub-scores 4 and 5 since it is the polymer with the highest T_g_. Regarding the migration risk sub-scores (1 and 2), PC exhibits acceptable values, while its score for permeation (score3) is moderate. In contrast, PAN exhibits very poor permeability to oxygen and acetic acid (and thus has a high score3) and moderate values for the other scores, and this is the reason for being among the highest-ranked polymers. It should be stressed that PAN and PS (ranked in third place) exhibit moderate to low values for sub-scores 4 and 5. This arises from the fact that the scores have been normalized with respect to the highest T_g_ (exhibited by PC). However, PS exhibits a T_g_ of more than 85 °C, which is sufficiently higher than the typical filling and storage temperature of vinegar. Consequently, no serious problems arising from the T_g_ of PS or PAN would be expected. In other words, the sub-scores may lead to misleading conclusions and must be carefully evaluated. PS exhibits the same moderate value for score3 as PC, but scores 1 and 2 are considerably higher than those of PC and PAN. Thus, based on the above and despite being in third place, PS seems to be one of the optimum choices. In addition, based on the above, a proper multi-layered composite film can be designed. For example, a PS film coated with PAN will exhibit very high values for all five scores, and PAN will improve (decrease) the permeability of the pure PS film. Moreover, based on scores 1 and 2, it is predicted that migration into a hydrophilic food (such as vinegar) will be lower in the case of PS compared to PVA. This was confirmed experimentally using olive oil as migrant, as discussed above and presented in Table 2.

In Table 4, the ranking of 22 polymers for their suitability to be used as packaging materials for ketchup is presented. It should be mentioned that for ketchup, a hot filling process (88 °C) [23] was assumed. Thus, in contrast to the case of vinegar, in which the storage temperature and maximum exposure temperature are identical, these two temperatures do not coincide for ketchup. Consequently, score4 and score5 also do not coincide for the case of ketchup (whereas in the case of vinegar, they were identical). Since both ketchup and vinegar are of a hydrophilic nature it is not surprising that the ranking is similar. Again, PC and PAN are in the first positions. PS is in a slightly lower position (position 5). The main reason for this is the very low score5. This arises from the fact that the T_g_ of PS is very close to hot filling temperature. Thus, PS should be excluded. For the same reason, and despite being in the second position, PAN should also be excluded since it also exhibits a low score5. Poly(vinyl pyrrolidone) (PVP) can also be excluded. The main reason for its high total score is its high T_g_, which results in very high values for score4 and score5, but the values for the other scores are low. Based on the above, it seems that PC and poly (ethylene terephthalate) (PET) are the most suitable materials for ketchup. It is worth mentioning that the negative values for scores 4 and 5 arise from the fact that these polymers exhibit T_g_ values lower than the storage and maximum exposure temperatures, respectively.

In Table 5, the ranking of 22 polymers for their suitability to be used as packaging materials for olive oil is presented. PC and PVP are again in the first positions due to the high scores 4 and 5 (high T_g_ values). However, the values for the other scores are quite low. Although PAN and PVA exhibit low scores 4 and 5, their T_g_ values are sufficiently higher than the storage and exposure temperatures of olive oil; thus, the effect of these scores can be neglected. Both PVA and PAN exhibit very high values for scores 1, 2 and 3 and seem to be the best options.

Based on the above, the proposed methodology can be considered as a decision-support tool to be used by experts. The overall score can be used to exclude polymers that are surely unsuitable for a given food. However, as discussed above, the final material selection cannot be based simply on the overall score, and the sub-scores should be taken into account. A major limitation/drawback that could be addressed is the use of T_g_ without accounting for the degree of crystallinity. Various semi-crystalline polymers exhibit rather high degree of crystallinity; thus, the influence of T_g_ (amorphous regions) would be less intense. For such polymers, the melting point could also be considered. This limitation is the major reason that common polymers widely used in food packaging, e.g., poly(propylene) (PP), are ranked at low positions (PP exhibits a sub-zero T_g_) and receive negative respective sub-scores. Additionally, another limitation/drawback of the proposed methodology is that the sub-scores are normalized with respect to the minimum or maximum values observed among the candidate polymers. If a new polymer is included among the candidate polymers, the sub-scores will change if this polymer presents a maximum or minimum value. In such a case, the sub-score of all other polymers will change accordingly, and some sub-scores, e.g., score1, will change proportionally. Thus, no serious alteration of the ranking should be expected. Of course, if many more polymers are included, then this is not the case. This could be addressed by using an ideal (non-existing) polymer with a very high T_g_, very low oxygen permeability, etc., as a reference. For the permeability to flavor compounds, this is not possible. Finally, exclusion criteria could be applied before calculating the final overall score and ranking. The use of exclusion criteria, the use of fixed reference values and the consideration of the degree of crystallinity could make the proposed tool accessible to non-experts and hopefully will be presented in future work.

Finally, it is worth mentioning that the methodology could be potentially applied to any polymer (synthetic, natural, etc.) provided that reliable values for HSPs are available. Here, the polymers mentioned on the HSP official webpage [9] were used. In many natural polymers, e.g., cellulose and chitin, the solubility is extremely limited in the vast majority of solvents, and thus the derivation of reliable HSP values is hindered (HSPs are typically determined by solubility tests). For this reason, no such polymers were included in this study. Group contribution methods are available for theoretically estimating the HSP of polymers [10], but these predictions should be treated with care. 

## 4. Conclusions

A simple methodology was proposed for polymer screening for food packaging applications. The methodology accounts for migration risk, the permeability of the polymer to oxygen and volatile flavor compounds, as well as the T_g_ of the polymer and the storage and the maximum exposure temperature of the food. The polymers are ranked according to their total score, which is derived as the sum of various sub-scores. The sub-scores are much more important than the total score and can assist in recognizing the advantages and disadvantages of each polymer for a given food. The methodology can assist in finding proper candidate materials, exclude polymers that would be clearly unsuitable for a given food or even design multi-layered composites with improved properties.

One of the basic assumptions of the proposed methodology is that the polymer–food interaction can act as an indirect measure of the migration risk. This seems to be preliminarily supported by migration tests from PVA and PS films in two food simulants. The migration was tested in two food simulants that are proposed in the EU regulation regarding plastic materials in contact with foods. The above conclusion is rather important, since in the EU regulation the food simulants are proposed based on their potential interaction with the migrants. Specifically, in the EU regulation, it is mentioned that there are three hydrophilic simulants (A, B and C) that can extract hydrophilic substances (migrants) and, correspondingly, there are two hydrophobic simulants (D1 and D2) that can extract hydrophobic migrants. There is no doubt that the food–migrant interaction is important, but the insights of this work suggest that the polymer–food interaction could be additionally considered for accounting for the migration risk. Of course, further migration experiments with more migrants and in more food simulants should be performed in order to provide a better and more thorough understanding of the migration process.

## Figures and Tables

**Table 1:** Thickness of the films with and without olive oil that were used for the migration tests in food simulants A and D1.

Polymer	Film Thickness, mm	
	Simulant A	Simulant D1
PS (1st replicate)	0.20	0.20
PS (2nd replicate)	0.31	0.30
PVA (1st replicate)	0.21	0.35
PVA (2nd replicate)	0.35	0.36
PS-olive oil (1st replicate)	0.34	0.29
PS-olive oil (2nd replicate)	0.30	0.28
PVA-olive oil (1st replicate)	0.22	0.20
PVA-olive oil (2nd replicate)	0.44	0.18

**Table 2:** Overall % mass loss of olive oil-polymer and % mass loss of neat polymer films in two food simulants. The difference between the average values of the two mass losses is presented as the % mass loss of olive oil.

Polymer	Simulant	
PS	A	D1
Overall % mass loss	1.5 ± 0.3	1.9 ± 0.3
% mass loss of neat polymer	1.6 ± 0.5	1.5 ± 0.4
% mass loss due to olive oil migration	0.0	0.4
PVA		
Overall % mass loss	2.8 ± 0.1	1.3 ± 0.1
% mass loss of neat polymer	1.8 ± 0.1	1 ± 0.3
% mass loss due to olive oil migration	0.9	0.3

**Table 3:** Polymer ranking for their suitability to be used as packaging material for vinegar.

Ranking	Polymer	Total Score	Score 1	Score 2	Score 3	DevScore 3	Score 4	Score 5
1	polycarbonate	4.32	0.64	0.64	0.52	0.08	1.00	1.00
2	polyacrylonitrile	4.31	0.53	0.54	0.96	0.06	0.66	0.66
3	polystyrene	3.92	0.80	0.81	0.58	0.29	0.57	0.57
4	polyethylene terephthalate	3.90	0.63	0.64	0.53	0.08	0.79	0.79
5	polyvinylpyrrolidone	3.79	0.38	0.40	0.54	0.26	0.97	0.97
6	polyvinyl chloride	3.79	0.70	0.71	0.65	0.20	0.54	0.54
7	polyphenyleneoxide	3.37	0.69	0.70	0.50	0.08	0.49	0.49
8	polymethyl methacrylate	3.33	0.58	0.59	0.63	0.08	0.46	0.46
9	polyvinyl alcohol	3.03	0.26	0.28	0.79	0.29	0.45	0.45
10	polyethyl methacrylate	2.99	0.63	0.64	0.57	0.12	0.29	0.29
11	polyvinylbutyral	2.82	0.56	0.57	0.58	0.09	0.27	0.27
12	polypropylene	2.82	1.00	1.00	0.60	0.47	−0.19	−0.19
13	poly lactic acid	2.75	0.56	0.57	0.60	0.06	0.21	0.21
14	polyvinyl acetate	2.67	0.84	0.84	0.51	0.30	−0.02	−0.02
15	polyurethane	2.43	0.62	0.63	0.58	0.12	0.01	0.01
16	ethylene vinyl alcohol	2.13	0.31	0.33	0.53	0.31	0.22	0.22
17	polypropylene oxide (poly propylene glycol)	1.35	0.55	0.56	0.46	0.03	−0.34	−0.34
18	polyvinylidenefluoride	0.74	0.35	0.37	0.54	0.18	−0.53	−0.53
19	polycaprolactone	0.54	0.62	0.63	0.48	0.02	−0.84	−0.84
20	polyethylene	0.32	0.93	0.92	0.50	0.41	−1.27	−1.27
21	polyethylene oxide (poly ethylene glycol)	0.20	0.59	0.60	0.53	0.06	−1.03	−1.03
22	polyoxymethylene	0.14	0.45	0.46	0.48	0.14	−0.86	−0.86

**Table 4:** Polymer ranking by suitability to be used as packaging materials for ketchup.

Ranking	Polymer	Total Score	Score 1	Score 2	Score 3	DevScore 3	Score 4	Score 5
1	polycarbonate	4.15	0.65	0.65	0.42	0.13	1.00	1.00
2	polyacrylonitrile	3.77	0.54	0.54	0.83	0.19	0.66	0.36
3	polyvinylpyrrolidone	3.68	0.39	0.40	0.49	0.21	0.97	0.94
4	polyethylene terephthalate	3.52	0.64	0.65	0.42	0.14	0.79	0.60
5	polystyrene	3.52	0.82	0.82	0.56	0.20	0.57	0.19
6	polyvinyl chloride	3.19	0.72	0.72	0.54	0.24	0.54	0.13
7	polyphenyleneoxide	2.85	0.70	0.70	0.47	0.07	0.49	0.03
8	polyvinyl alcohol	2.71	0.25	0.27	0.90	0.21	0.45	−0.05
9	polymethyl methacrylate	2.57	0.60	0.60	0.47	0.27	0.46	−0.04
10	polyethyl methacrylate	2.21	0.66	0.66	0.49	0.20	0.29	−0.36
11	polyvinylbutyral	1.99	0.56	0.57	0.49	0.18	0.27	−0.39
12	polypropylene	1.97	1.00	1.00	0.72	0.32	−0.19	−1.28
13	polyvinyl acetate	1.85	0.85	0.85	0.56	0.19	−0.02	−0.95
14	poly lactic acid	1.73	0.58	0.58	0.43	0.28	0.21	−0.50
15	ethylene vinyl alcohol	1.55	0.33	0.34	0.57	0.19	0.22	−0.49
16	polyurethane	1.33	0.65	0.65	0.46	0.23	0.01	−0.90
17	polypropylene oxide (poly propylene glycol)	0.08	0.58	0.58	0.42	0.08	−0.34	−1.56
18	polyvinylidenefluoride	−0.74	0.39	0.39	0.47	0.13	−0.53	−1.93
19	polycaprolactone	−1.25	0.64	0.64	0.41	0.09	−0.84	−2.51
20	polyethylene	−1.49	0.93	0.93	0.62	0.28	−1.27	−3.32
21	polyoxymethylene	−1.71	0.47	0.48	0.38	0.14	−0.86	−2.56
22	polyethylene oxide (poly ethylene glycol)	−1.74	0.62	0.62	0.47	0.16	−1.03	−2.88

**Table 5:** Polymer ranking for their suitability to be used as packaging material for olive oil.

Ranking	Polymer	Total Score	Score 1	Score 2	Score 3	DevScore 3	Score 4	Score 5
1	polyacrylonitrile	5.13	0.92	0.92	0.96	0.04	0.66	0.70
2	polyvinyl alcohol	4.82	1.00	1.00	0.93	0.09	0.45	0.51
3	polyvinylpyrrolidone	4.16	0.60	0.60	0.51	0.20	0.97	0.97
4	polycarbonate	3.45	0.33	0.33	0.40	0.09	1.00	1.00
5	polyvinyl chloride	3.21	0.47	0.47	0.57	0.14	0.54	0.59
6	polymethyl methacrylate	3.12	0.53	0.53	0.54	0.12	0.46	0.51
7	polyethylene terephthalate	3.10	0.35	0.35	0.40	0.09	0.79	0.81
8	ethylene vinyl alcohol	3.07	0.69	0.69	0.59	0.11	0.22	0.30
9	polystyrene	2.86	0.33	0.33	0.51	0.16	0.57	0.62
10	polyvinylbutyral	2.57	0.49	0.49	0.49	0.16	0.27	0.35
11	poly lactic acid	2.54	0.50	0.50	0.51	0.10	0.21	0.29
12	polyethyl methacrylate	2.51	0.46	0.46	0.48	0.16	0.29	0.36
13	polyphenyleneoxide	2.34	0.27	0.27	0.39	0.09	0.49	0.54
14	polyurethane	1.99	0.46	0.46	0.48	0.14	0.01	0.11
15	polypropylene	1.59	0.31	0.31	0.62	0.25	−0.19	−0.07
16	polyvinyl acetate	1.34	0.19	0.19	0.45	0.17	−0.02	0.08
17	polyvinylidenefluoride	1.29	0.60	0.60	0.50	0.10	−0.53	−0.38
18	polypropylene oxide (poly propylene glycol)	0.93	0.40	0.40	0.34	0.13	−0.34	−0.21
19	polyoxymethylene	0.17	0.47	0.47	0.39	0.11	−0.86	−0.67
20	polyethylene oxide (poly ethylene glycol)	−0.10	0.45	0.45	0.43	0.16	−1.03	−0.82
21	polycaprolactone	−0.20	0.30	0.30	0.35	0.09	−0.84	−0.65
22	polyethylene	−0.99	0.16	0.16	0.49	0.22	−1.27	−1.03

## Data Availability

The original contributions presented in this study are included in the article. Further inquiries can be directed to the corresponding author.
